# Serum Testosterone Level, Testosterone Replacement Treatment, and Prostate Cancer

**DOI:** 10.1155/2013/275945

**Published:** 2013-09-18

**Authors:** Ali Atan, Altug Tuncel, Suleyman Yesil, Derya Balbay

**Affiliations:** ^1^Gazi University School of Medicine, Department of Urology, Besevler, 06125 Ankara, Turkey; ^2^Ministry of Health, Ankara Numune Research and Training Hospital Third Department of Urology, Sihhiye, 06120 Ankara, Turkey; ^3^Sisli Memorial Hospital, Department of Urology, 34120 Istanbul, Turkey

## Abstract

There has been an increase in the number of individuals seeking testosterone (T) replacement treatment (TRT) due to a decrease in their blood T levels. Prostate cancer (PCa) is also an important issue in the same age group. However, we, urologists, are anxious about PCa development after T treatment. This is because it has been assumed that T may cause PCa or exacerbate insidious PCa which is already present. In this paper, recent developments regarding the relationship between serum levels of sex hormone and prostate tissue, the causal relationship between T and development of PCa, the effect of TRT on the group of patients who are at high risk of developing PCa, the suitability of TRT for patients who have already been diagnosed with PCa, and the effect of TRT on serum prostate-specific antigen level are analyzed.

## 1. Introduction

According to the Bureau of Census of the United States, the population of men over the age of 65 that is 35 millions in year 2000 will reach 40, 54.6, and 70 millions by years 2010, 2020, and 2030, respectively [1, 2]. According to another study conducted in Germany, it is estimated that 1/3 of the population will be over 60 years and that 11% will be over 80 years of age by year 2050 [3]. Serum testosterone (T) level in men has been shown to be decreased significantly with aging in both cross-sectional and longitudinal studies [4–7]. Therefore, the number of patients needing T replacement treatment (TRT) due to a decrease in their T levels secondary to aging is increasing. Despite the accumulating knowledge on the effects of T, many still continue to carry a suspicion on the use of T. Until recently, regarding prostate cancer (PCa), TRT has been indisputably considered as adding fuel to the flames or feeding a hungry tumor [8]. It was scared that TRT will cause PCa or flare up occult PCa which stemmed from the studies of Huggins and Hodges in 1941 [9]. According to their work, PCa regressed when serum T levels had been reduced and progressed when exogenous T had been given. However, there has been no clear evidence reported since then that men receiving or with high T levels have had an increased risk of developing PCa [8, 10]. On the contrary, in a large prospective study investigating the correlation between high levels of circulating T and risk of PCa, higher levels were found to be related to reduced risk of PCa [11]. Additionally, there have been several contemporary reports stating that decreased serum T levels can be a marker for occult PCa [12]. PCa has a much more aggressive course regardless of disease stage in patients with decreased serum levels of T [13, 14], and PCa patients with low serum T levels do not do well with the treatment [15–17]. Mearini et al. reported that the patients have the increased probability of finding advanced disease when the patients who underwent radical retropubic prostatectomy have lower preoperative serum T levels [18]. Given these data, it seems that low T level is a risk factor in the development, diagnosis, and treatment of PCa.

 Some studies on the effects of T have revealed that T is not only a sex hormone but instead it also has many positive effects on other body functions [19, 20]. Diabetes mellitus, development of metabolic syndrome, increased risk of death due to cardiovascular diseases, and pathologic bone fractures due to severe loss of bone are well-known negative effects of iatrogenic or spontaneous T depletion [21, 22]. A constellation of symptoms known as late-onset hypogonadism characterized with sexual dysfunction (erectile dysfunction and loss of libido), deterioration of cognitive functions (decreased intellectual capacity, depressive mood changes, irritability, and loss of concentration), sleep disorders, loss of muscle mass and strength, increase in visceral fatty tissue, hair loss, skin changes, decrease in bone mineral density, osteopenia, and increase in development of fractures is seen in aging males [3, 23]. The real problem is the increased incidence of PCa in the same age group of men. 

 Therefore, we will try to clarify the following topics according to the present data.

1. How does prostate tissue interact with serum T?
2. Is there any relationship between serum T level and PCa?
3. Does TRT have any role in the development of PCa?
4. Can patients who are at high risk of developing PCa be placed on TRT?
5. Can patients with diagnosis of PCa be placed on TRT?
6. Timing of TRT in patients who have also received treatment for PCa is highlighted.
7. TRT and serum prostate-specific antigen (PSA) level are discussed.

*(1) How Does Prostate Tissue Interact with Serum T?* Sex hormones exert their effects at the periphery after binding to androgen receptors. T saturation in the prostatic tissue is extremely low. Studies carried out on human, rat, and dog prostates have shown that serum T level required for maximum androgen receptor binding is as low as 2–4 nM (60–120 ng/dL) [24, 25]. It is clear that this serum level is approximately half the level of T used in diagnosing late-onset hypogonadism [23]. This finding indicates that prostate tissue is saturated with T even if its serum levels are extremely low, and this is explained by the saturation theory. According to this, serum T levels above this should not have any significant effect on the prostatic tissue [8]. This assumption is also supported by a randomized, placebo-controlled clinical study. This study showed that intraprostatic levels of T and dehydro-T and cell proliferation markers did not change although serum T level increased after exogenous T treatment in comparison with the placebo group. These data have indicated that there is no direct correlation between serum T levels and intraprostatic hormonal milieu [26].

*(2) Is There Any Relationship between Serum T Level and PCa?* PCa development has been reported to be linked with increased serum T levels in some studies [27, 28]. However, not all studies where correlation between serum T levels and PCa development was looked for resulted in similar conclusions, simply because there is no such correlation. 

 Incidence of PCa was reported to be similar in patients with normal serum T levels and patients with hypogonadism. After prostate biopsy, PCa was diagnosed in 11 out of 77 men (14.2%) with hypogonadism, normal digital rectal examination findings, and serum prostate-specific antigen (PSA) < 4 ng/mL [12]. In a more recent study published by the same author, PCa was diagnosed in 15.1% of 345 hypogonadal patients with serum PSA levels <4 ng/mL, after prostatic biopsy [29]. These results are in accordance with those of PCa prevention trial where the incidence of PCa were found in 15.2% of the patients in the placebo arm [30]. 

 Roddam et al. analyzed 18 studies where correlation between PCa and sex hormones was investigated [31]. In this analysis, 3886 patients with PCa and 6438 control patients were evaluated. As a result, no correlation between serum sex hormone levels and development of PCa was found. Data from this analysis showed that initial serum T levels were not high in patients who developed PCa later in their followups, and also there is no increased risk of developing PCa in patients with initially high serum levels of T compared with those with lower initial serum T level. 

 There has been an inverse correlation between the age of the patient when PCa is developed and serum T level. The incidence of PCa is the lowest during 2-3 decades when serum T levels were at their highs. Inversely, PCa is diagnosed at significantly higher rates after the 6th decade when serum T levels started to decrease towards their nadir levels [8]. These data indicate that serum T levels do not have a significant role in the development of PCa. 

*(3) Does TRT Have Any Role in the Development of PCa?* This is the most fearful complication attributed to TRT. There have been some reports indicating PCa development after TRT [32, 33]. However, there was no clear evidence in any of these reports proving that TRT definitely results in PCa. Many more reports are in contradiction to those ones. In one of the past reports, no PCa development was seen in patients who received TRT up to 10 years [34]. Two studies reported that there is no overall increase of developing prostate cancer with TRT [35, 36]. In another study, only one patient developed PCa among the 54 who received TRT for symptomatic hypogonadism for 30 months [37]. In a review of 7 studies published by Rhoden and Morgentaler, the incidence of PCa was reported to be only 1.1% among the 461 patients who received TRT for 6 to 36 months [38]. Additionally, a meta-analysis on the comparison of TRT with placebo revealed equal negative effects on the prostate [39]. In another study by McLaren et al., the incidence of PCa was calculated as around 4% after 2 years of TRT [40]. Shabsigh and coworkers published a paper where they reviewed 44 studies [41]. PCa developed in 7 (1.3%) out of the 542 patients who received TRT, whereas 5 (1.5%) out of the 333 patients in the placebo arm developed PCa. In one of the nonplacebo-controlled studies, 20 men developed PCa generally after 2 years of TRT [33]. In other nonplacebo-controlled studies, PCa incidence was reported to be between 1.2% and 4.5% [41]. In a recent study including 1365 patients on long-term TRT, PCa developed only in 14 patients. Mean time of TRT in the study was 6.3 years. Diagnosis of PCa was mostly due to significant elevation of serum PSA level. Although 12 patients had the Gleason grade <4 and clinical stage T1c PCa and only 2 patients had high-risk PCa, all of the PCa patients were suitable for curative treatment. According to their results, the authors conclude that TRT in men with androgen deficiency seems safe when carefully and regularly monitored [42]. The present data do not support an unequivocal correlation between PCa and TRT. A recent review states that although no controlled studies have yet been performed and there is a paucity of long-term data, the available literature strongly suggests that TRT neither increases the risk of PCa diagnosis in normal men nor causes cancer recurrence in men who were successfully treated for PCa [43].

*(4) Can Patients Who Are at High Risk of Developing PCa Be Placed on TRT?* There has been an increasing trend in using transrectal prostate biopsy procedures after the widespread use of PSA which naturally resulted in increasing diagnosis of prostatic intraepithelial neoplasia (PIN) and atypical small acinar proliferation (ASAP) independent of PCa. The patients with these lesions are considered to be at high risk of developing PCa. There is only one study published in the international literature investigating whether T replacement treatment causes an increase in PCa in men with high-grade PIN in their first set of prostate biopsies. Rhoden and Morgentaler prescribed T replacement treatment to 50 men with benign prostate hyperplasia and 20 men with high-grade PIN in their prostate biopsies for 1 year. No significant increase in serum PSA levels was reported in both groups after treatment. Only one patient developed PCa in the high-grade PIN group, and investigators concluded that presence of high-grade PIN is not a contraindication for TRT [44]. Lefkowitz et al. found that rate of PCa was 26% in patients with high grade PIN who were not given TRT [45]. There has been no study on ASAP, and currently we do not have enough data on the effects of TRT in patients with high-grade PIN and ASAP. Therefore, we should continue to be careful with TRT in such patients. 

*(5) Can Patients with the Diagnosis of PCa Receive TRT?*

*(a) TRT in Patients with Untreated PCa.* There has been no concrete evidence as of yet. One case report and two studies including 13 and 7 patients have been published on TRT in patients with PCa but did not receive any treatment for it. In the case report, an 84-year-old man with a serum PSA level of 8.1 ng/mL and a PCa of a Gleason score of 3 + 3 received TRT for 2 years for symptomatic hypogonadism [46]. As symptoms of hypogonadism regressed, his serum PSA level went down to 5.1 ng/mL with no evidence of clinical progression of the PCa. The author concluded that this is an important observation which interrogates our perception of the correlation between PCa and TRT despite the fact that this was made only on a single patient. The same author presented his observation on 13 patients with symptomatic hypogonadism and concomitant PCa who elected to go with active surveillance for PCa but received TRT for their hypogonadism [47]. All patients were evaluated with serum PSA checks and repeat prostate biopsies. Median duration of TRT was 2.5 years. The mean age was 58.8 years. Mean serum PSA level and prostate volume did not change with TRT. No local progression and distant metastasis were observed with TRT. These results seem contradictory to the traditional belief that the men with untreated PCa are never given TRT. This study is important for its being the first one carried out on TRT in patients who did not receive active treatment for PCa. In another study by Morales, efficacy of TRT was evaluated in 7 hypogonadal men with untreated PCa. The mean age of the patients was 68 years. Three patients received long-term (33 months, 96 months, and 51 months) TRT. None of the patients had metastatic diseases during TRT even if serum PSA level increased [48].

 The present data are not mature enough to recommend TRT routinely for symptomatic hypogonadism in patients with PCa, but they are encouraging. In accordance with the guidelines, we currently take into consideration that no recommendation even with the present new data could be done on TRT in PCa patients who are not treated for it [23, 49]. 

*(b) TRT in Patients Who Received Treatment for PCa.* Such patients are much more important to us. As a result of the increasing number of prostate biopsies, many more patients are diagnosed with PCa. Since local treatment modalities for localized PCa are successful, prolonged survival rates have been achieved. With advanced age of the patient, another health problem, namely, late-onset hypogondism, emerges. Contemporary studies try to elucidate whether TRT can be given for symptomatic hypogonadism after definitive treatment for PCa. Recent studies provide some new evidence that TRT is no more dangerous as it was thought to be in the past and should therefore be withhold.

 The first study on TRT for symptomatic hypogonadism developed in men who were treated for PCa in the international literature appeared in 2004. T treatments were given to 7 patients after radical prostatectomy for 24 months, and neither clinical nor biochemical progression was seen in any patient [50]. A year after publishing this series, another study on 10 patients who received TRT after radical prostatectomy for 1 year was carried out. The mean patient age was 64 years. Mean followup after the treatment was 19 months, and no patients recur with serum PSA elevations in their followups [51]. Mulhall et al. also treated 22 men with symptomatic hypogonadism after radical prostatectomy with TRT [52]. T gel treatment was started for a mean of 26 months after radical prostatectomy and mean followup after T treatment was 24 months. Only one patient (4.5%) with a Gleason score of 8 had reccurrence at the 12th month of TRT and 17 months after radical prostatectomy. First of the larger series was published by Khera et al., where they investigated the effects of TRT when given for hypogonadism in 57 patients after radical prostatectomy [53]. Mean time elapsed after radical prostatectomy was 36 months and serum PSA levels were <0.1 ng/mL in all patients. The mean follow-up time after T treatment was 13 months. No patients had serum PSA recurrence after the treatment. In a multicenter study presented at the 2010 American Urological Association Annual Meeting, 69 patients received TRT after radical surgery [54]. TRT was started at a mean of 24 months and lasted for at least 6 months. There was no biochemical recurrence in a mean follow-up time of 19 months.

The conclusion from all of these studies is that T replacement treatment can be given to those patients who underwent radical prostatectomy for cT1-2, a Gleason score of 7, and serum PSA level of 10 ng/mL PCa and who had negative surgical margins and no lymph node involvement, after achieving a postoperative nadir serum PSA level. Nevertheless, these patients should be thoroughly informed about TRT, written consent should be obtained, and patients should be monitored closely after the treatment.

 In a new study, 103 patients were treated with TRT. Of these patients, 23 were high-risk patients (Gleason score ≥ 8, positive surgical margin or lymph node involvement). None of the patients either in the high-risk group or in the whole group progressed in the 27.5 months of followup. This study is important in the sense that even high-risk patients, for recurrence, can be given TRT when needed [55]. Obviously, the present data are not enough to rationalize T replacement treatment in the high-risk group of patients and must be supported with other studies.

 Leibowitz et al., retrospectively, studied 96 patients who received TRT for symptomatic hypogonadism after treatment of PCa [56]. Of these patients, 59 were treated with androgen deprivation, 24 patients underwent radical prostatectomy, 12 patients received radiotherapy, and 1 patient was treated with brachytherapy. Mean serum PSA level was 0.1 ng/mL, and mean duration of T treatment was 15 months. Serum PSA elevation was observed in 41 patients in whom radiologic evidence of progression was also present. There was no change in serum PSA levels in the remaining 55 patients. TRT was continued without any interruption for 36 months in 31 patients. It was concluded that radical prostatectomy as the primary treatment and addition of dutasteride to TRT were the two factors which prevented elevation of serum PSA level, whereas high pretreatment serum PSA level was found to be associated with PSA progression. The authors also concluded that increments in serum T levels do not cause disease progression, that PSA increase after TRT does not necessarily translate into clinical or symptomatic disease progression, and that TRT improves symptoms of hypogonadism and can be continued for years.

 It is generally accepted that there is no residual prostate tissue if serum PSA level reaches nadir levels after radical prostatectomy and that TRT is thought to be safe if symptomatic hypogonadism develops according to the current new data. But such thought is arguable after radiotherapy, since serum PSA level is detectable in the majority of such patients and what is going to happen if T treatment is started is unpredictable. Only four studies published in the international literature on this topic are available [22, 57–59]. In the first study, 31 patients who had been treated with brachytherapy for PCa were given TRT for symptomatic hypogonadism. Of these, 20 patients had been treated with brachytherapy only, and the remaining 11 patients had also received external beam radiotherapy in addition to brachytherapy. TRT was started 6 months after the treatment at the earliest. Patients were evaluated every 6 months for the first 5 years, and the annually thereafter. The mean patient age was 65 years, and the duration of T treatment was 4.5 years. Being <1 ng/mL in all patients, serum PSA levels were <0.1 ng/mL in 23 (74.2%) and <0.5 ng/mL in 30 (96.7%) patients. The author has concluded that TRT does not cause PCa recurrence or progression after brachytherapy [57]. Another study included 5 patients with symptomatic hypogonadism who received external beam radiotherapy for PCa. The mean patient age was 65. These patients received TRT for a mean of 14.5 months. None of the patients had serum PSA elevation due to the treatment or recurred with PCa [22]. Davila et al. also compared the effect of TRT after surgical treatment and radiotherapy [58]. TRT was given to 8 patients after open surgeries and to 6 patients after laparoscopic surgeries in addition to 6 patients treated with external beam radiotherapy. Patients treated by either open or laparoscopic surgeries were grouped as A, and others treated by external beam radiotherapy were grouped as B. Serum PSA levels before TRT were 0.1 in group A and 0.15 in group B, and PSA levels at 12 months of TRT were 0.1 in both groups. These authors concluded that TRT can be given for symptomatic hypogonadism after both surgery and radiotherapy. In a new study by Pastuszak et al., 13 patients with PCa who were treated with brachytherapy or external beam radiotherapy were evaluated. Median followup after TRT was 29.7 months. There was no significant increase in serum PSA level. No patient had any recurrences of PCa after TRT [59]. 

The present data are encouraging clinicians to prescribe T in the presence of symptomatic hypogonadism after external beam radiotherapy, albeit still immature. Such patients may receive TRT after all aspects of this treatment are discussed with the patient and written consent was obtained. These patients should be also closely followed up with serum PSA measurements and digital rectal examination.

*(6) Timing of TRT in Patients Who Had Received Any Treatment for PCa Previously.* Timing of TRT when symptomatic hypogonadism develops in patients who received curative treatment for localized PCa is still debatable. Serum PSA reaches its nadir value shortly after radical prostatectomy, and TRT can be instituted thereafter [53]. The European Association of Urology Guidelines recommends to begin TRT in first year after the surgery [60]. This issue is not as clear after radiotherapy yet, because prostatic tissue remains after radiotherapy and serum PSA may not reach its nadir level. For this reason, T treatment can be given when serum PSA level is <1 ng/mL [22]. 

*(7) TRT and Serum PSA Level.* One of the important points is the effect of TRT on serum PSA level. TRT causes minor PSA increase [37, 40]. According to these data, hypogonadal men without prostate cancer will have a detectable but slight increase in serum PSA level during TRT. Excessive serum PSA elevation during TRT should be evaluated for the possibility of prostate cancer. There is no correlation between endogenous T concentration and serum PSA level. Variation in endogenous T concentration within the physiological range does not appear to influence PSA levels [61]. Even in men who had supraphysiological T levels after exogenous T treatment, serum PSA levels did not show significant change [62, 63]. In a systematic review paper by Shabsigh et al., TRT does not cause significant elevation of serum PSA level [41].

 As a general agreement, serum PSA levels within normal range after TRT in hypogonadal patients should be controlled at the 3rd and the 12th months, then at each 6th or 12th months [64]. Serum PSA level should remain below 4 ng/mL. Increase of serum PSA level should be ≤1.5 ng/mL in a year or ≤0.75 ng/mL per year for 2 years. If serum PSA level increases above 4 ng/mL during TRT, prostatic biopsy should be recommended to the patients [65].

## 2. Conclusion

 PCa development is independent from endogenous serum T levels. TRT for symptomatic hypogonadism does not increase the risk of PCa development. Data derived from the current literature indicate that TRT can be given to symptomatic hypogonadal patients who received radical prostatectomy, brachytherapy, or external beam radiotherapy for PCa with detailed discussion, obtaining written consent and with close and vigilant followup. TRT can be started in first year after radical prostatectomy and with serum PSA level less than 1 ng/mL after external beam radiotherapy. Despite the fact that there is still no enough evidence to make a general recommendation, the current data are encouraging. We need to be careful until multiple studies with longer followups which support the current data are available.
